# Psychometric evaluation of the Chinese version of the Scale of Effects of Social Media on Eating Behaviour and research of its influencing factors

**DOI:** 10.1186/s12889-024-17923-1

**Published:** 2024-02-17

**Authors:** Kaiyan Xu, Chunguang Liang, Ying Zhao, Fan Zhang, Chunyan Zhang, Yanhong Zhang, Yefan Zhang, Zhaoquan Jiang

**Affiliations:** 1 School of Nursing, Jinzhou Medical University, No. 40, Section 3, Songpo Road, Linghe District, Jinzhou, 121001 P.R. China; 2 Shenyang Jianzhu University Hospital, No. 25, Hunnan Middle Road, Hunnan District, Shenyang, 110168 P.R. China

**Keywords:** Social media, Eating behavior, College students, Psychometric properties

## Abstract

**Background:**

Social media has become an indispensable part of contemporary young people's lives, and the influence of social media on college students' eating and other health-related behaviors has become increasingly prominent. However, there is no assessment tool to determine the effects of social media on Chinese college students' eating behavior. This study aims to translate the Scale of Effects of Social Media on Eating Behaviour (SESMEB) into Chinese. Its applicability to Chinese college students was examined through reliability and validity indexes, and the influencing factors of SESMEB were explored.

**Methods:**

The questionnaire survey included 2374 Chinese college students. The Brislin translation model was used to translate the original scale into Chinese. Exploratory factor analysis (EFA) and confirmatory factor analysis (CFA) were used to test the construct validity of the scale, and the content validity of the scale was assessed through the content validity index. The internal consistency of the scale was assessed by calculating Cronbach's alpha coefficient, McDonald's Omega coefficient, split-half reliability, and test–retest reliability. Multiple stepwise linear regression analysis was performed to identify potential influences on the effects of social media on eating behavior.

**Results:**

EFA supported the one-factor structure, and the factor loadings of each item on this dimension were higher than 0.40. CFA showed good model fitness indexes. The content validity index of the scale was 0.94. The Cronbach's alpha coefficient and McDonald's Omega coefficient for the scale were 0.964, the split-half reliability coefficient was 0.953, and the test–retest reliability was 0.849. Gender, education, major, frequency of social media use, online sexual objectification experiences, fear of negative evaluations, and physical appearance perfectionism explained 73.8% of the variance in the effects of social media on eating behavior.

**Conclusions:**

The Chinese version of the SESMEB has good psychometric properties and is a valid measurement tool for assessing the effects of social media on college students' eating behavior. Subjects who were female, highly educated, non-medical, had frequent social media use, online sexual objectification experiences, fear of negative evaluations, and physical appearance perfectionism used social media to have a higher impact on eating behavior.

## Introduction

People use social media as a platform to share and showcase their personal lives and interact with others through features such as commenting, liking, and forwarding [1]. Today, social media is the primary channel by which people communicate and get information [2], especially among young people [3]. In China, the percentage of college students using social media services such as WeChat and QQ is as high as 21.4%, and 40% of the time college students use their cell phones is spent using social media [4], with an average of 10 h of weekly use [5]. In a socio-cultural context of focusing on health and pursuing an ideal body shape, body shape management, and healthy eating have become a topic of great concern for young people on social media, and the effects of social media on eating behavior have thus become a critical issue in today's society [6].

Daily diet is an unavoidable and important part of an individual's life. Eating behavior is influenced by physiological, psychological, and socio-cultural factors [7]. In current society, the image of the ideal slim body has permeated all aspects of online media, and when individuals use social media, they may perceive a gap between the ideal and the reality, which in turn leads to dissatisfaction with their body appearance, resulting in eating disordered attitudes or behaviors. Disordered eating behaviors or attitudes are defined as unhealthy or nonadaptive eating behaviors such as restrictive eating, binge eating, and vomiting [8]. Studies have shown that adolescent girls with high BMI and perceived stress from motherhood and media and young adult women who use the Internet are more likely to experience eating disorders [9, 10]. Nowadays, the media has become the most popular source of health and nutrition information. People get advice and opinions about eating and health issues through texts, photos, and videos posted on social media and undoubtedly believe the information posted on the Internet [11], resulting in unhealthy eating behaviors. Unhealthy eating behaviors can lead to a range of problems including nutritional deficiencies and gut flora dysbiosis [12]. All of these phenomena indicate the need for further research on the effects of social media on eating behaviors.

Many theories have attempted to explain the mechanisms of media influence on eating behavior. Based on social comparison theory, individuals' self-evaluations about their bodies arise from the process of comparing their body states with those of others [13]. Individuals exposed to social media compare themselves with the ideal body image that is flooded on the Internet, and the comparison process generates body dissatisfaction and changes in eating behavior. Individuals with higher intensity of social networking site use have higher levels of body dissatisfaction [14], and idealized body image influences individuals' eating behavior [15]. Furthermore, the triple influence model of sociocultural theory (family, peers, and media) also directly confirms that social media use influences an individual's eating behavior [16]. Social networking sites as media factors spread the culture of idealized body image and perfect appearance image to people, which significantly influences individuals' eating behavior. Studies have shown that viewing pictures of ideal body image in social software can have an impact on the amount of food an individual eats [17]. With the popularity of social media, social networking sites and platforms were full of marketing advertisements. Consumers can be forced to make unhealthy food choices due to celebrity or internet celebrity promotions [18]. Also, in today's world of growing obesity problems and body image prejudice, the pursuit of ideal body standards (lean bodies for women; muscular bodies for men) may result in an increased desire to consume unrelated foods. Given that social media may have an immeasurable impact on eating behavior, especially in countries and regions where social media are widely used, such as China [19, 20]. This phenomenon deserves to be assessed with specialized, objective, and valid tools and indicators.

Over the past few decades, multiple scales measuring eating behavior have been created, verified, and adjusted to different cultural settings. For example, the Dutch Eating Behaviour Questionnaire (DEBQ) [21], the Restrictive Eating Scale (RS) [22], and the Three Factor Eating Questionnaire (TFEQ) [23]. Although assessment scales for eating behavior are well-established and widely used, they do not assess the effects of social media on eating behavior, and it is unknown how the degree of effect is defined. To address this shortcoming, Alev Keser et al. for the first time developed the Scale of Effects of Social Media on Eating Behaviour (SESMEB) in Turkish as a valid and dedicated tool to assess the effects of social media on eating behavior and then translated it into English [24]. The scale contains 18 items, which is a small number of items compared to other scales, and the item questions are comprehensive and easy to answer. Its Turkish version has exceptional psychometric properties as demonstrated by satisfactory construct validity, internal consistency reliability, and test–retest reliability [24]. The SESMEB is a comprehensive, reliable, and valid measurement tool. However, whether this scale is directly applicable in the context of Chinese culture or after appropriate modification needs further reliability and validity analysis.

Studies have shown that women and girls with online objectification experiences are more likely to exhibit restrictive eating behaviors because their objectification experiences lead them to view their appearance from an observer's point of view, emphasizing their outward appearance over their inward characteristics, and thus restricting their eating [25, 26]. Online objectification experiences are an experience of extensive attention to one's appearance in an online environment [27]. The widespread use of social media can enrich an individual's online objectification experience. The worry and fear of downside or negative evaluations of others is known as the fear of negative evaluations [28]. Related studies have demonstrated that fear of negative evaluations is associated with the drive to be thin [29], and Hamann and Levinson's studies have shown that fear of negative evaluations is a predictor of restrictive eating and bulimia [30, 31]. Other studies have evidenced that fear of negative evaluations is positively associated with restrictive eating and eating disorders in Western women and Chinese adolescents [32, 33]. Physical appearance perfectionism refers to individuals setting and strictly adhering to unrealistically high standards of appearance and judging their self-worth by whether or not they meet those standards [34]. Several systematic reviews have shown that perfectionism is a risk factor for eating disorders [35, 36], and is significantly associated with neurotic eating disorders [37]. Stoeber's study found that physical appearance perfectionism explained 9% to 17% of the variance in eating disorders [38]. In the context of social media's emphasis on the importance of body appearance and the promotion of an ideal body image, online objectification experiences, fear of negative evaluations, and physical appearance perfectionism have all been linked to a perfect body image on social media. Individuals with these traits constantly monitor their body size and appearance, generating body dissatisfaction and poor body imagery, which in turn leads to poor eating behaviors.

In the study, we translated and cross-culturally adapted the SESMEB scale into Chinese and further evaluated its psychometric properties in a population of Chinese college students. Based on the above literature review, the present study hypothesized that the effects of social media on eating behavior would be related to sociodemographic characteristics and that the use of social media would have a greater impact on eating behavior in subjects with online objectification experiences, fear of negative evaluations, and physical appearance perfectionism. Therefore, we conducted a cross-sectional influence factor analysis of SESMEB.

## Methods

### Participants and study design

This is a cross-sectional study that was conducted from October 30, 2023, to November 10, 2023, among college students in selected universities in Liaoning, Heilongjiang, and Shandong provinces using a convenience sampling method. The online survey was conducted through the Chinese data collection platform "Questionnaire Star". All participants signed an informed consent form. After 136 invalid questionnaires were excluded from the total of 2510 collected, a final sample of 2374 was obtained, providing an effective response rate of 94.58%. Inclusion criteria: (1) age 18–30 years old; (2) participants in this study gave their consent. Exclusion criteria: (1) questionnaire answering time less than 200 s; (2) identical responses to different questions; (3) students majoring in food and nutrition-related fields. The survey was made anonymous, but fifty participants were asked to leave their contact information so that the test–retest reliability of the scale could be assessed two weeks later. This study complied with the Helsinki Declaration's ethical principles [39] and the ethical standards of Jinzhou Medical University's Ethics Committee (Grant Number: JZMULL2021009).

### Translation and cultural adaptation

Professor Alev Keser authorized us before we translated and validated it. The forward–backward translation process was used, according to the Brislin translation [40]. First, two nursing graduate students independently translated the English version of the SESMEB into two different Chinese versions, followed by a third graduate nursing student who did not participate in the translation to compare the differences between the two versions, and convened all the translators and researchers to discuss and synthesize the comparisons, resulting in the first draft of the Chinese version. The first draft of the Chinese version was back-translated by two English experts (one university English teacher and one postgraduate in English interpretation) who had not seen the original scale, and two back-translated versions were obtained. Finally, the researchers discussed and compared the original scale, the first draft of the Chinese version, and the back-translated English version of the scale, and modified the controversial items, with a focus on linguistic and cultural adaptations, to make the scale more compatible with the Chinese language environment. Thirty college students were randomly selected to conduct a preliminary test of the scale. The final revision of the scale was made based on the participant's understanding of the content of the scale, their feelings, and suggestions during the process of filling out the scale, resulting in the final Chinese version of the SESMEB.

### Instruments

#### Questionnaire for general information

Include the participant's gender, age, grade, education, major, ethnicity, religious beliefs, being an only child, body mass index (BMI), and frequency of social media use. We defined never, < 1 h/d, 1–3 h/d, 3–5 h/d, and > 5 h/d as never, seldom, sometimes, often, and always using social media, in that order.

#### The Scale of Effects of Social Media on Eating Behaviour (SESMEB)

The scale was originally developed by Prof. Alev Keser et al. in 2018 to measure the effects of social media on the eating behavior of college students [24]. The SESMEB has one subscale and 18 items, which are five-point Likert scoring, with the scores of "always," "often," "sometimes", "seldom", and "never" in order of 5, 4, 3, 2, and 1 points, for a total score of 18 to 90. No substance has reverse coding. Higher scores indicate that participants' eating behaviors are more influenced by social media. The English version of the SESMEB has good reliability and validity with a Cronbach's alpha coefficient of 0.928 [24].

### Dutch Eating Behaviour Questionnaire(DEBQ)

DEBQ was developed by Van Strien et al. in 1986 and includes three subscales, Restrained Eating, Emotional Eating, and External Eating, which have been widely used in several countries [21, 41]. There are 33 question items in all, which are five-point Likert scoring. The Restrained Eating subscale and External Eating subscale have 10 items each, and the Emotional Eating subscale has 13 items. The External Eating subscale was chosen for this study to measure the degree of external eating in individuals, which has been validated in a group of Chinese adolescents, with a Cronbach's alpha coefficient of 0.874 [41], and the Cronbach's alpha coefficient in this study was 0.925.

### Online Sexual Objectification Experience Scale(OSOES)

The scale was developed by Chinese scholars Luo Yijun et al. in 2019, with a Cronbach's alpha coefficient of 0.82 [27]. It is suitable for measuring online sexual objectification experiences for all groups. The scale has a total of six items, which are five-point Likert scoring. Higher scores suggest that an individual has richer online sexual objectification experiences. The scale used in this study had a Cronbach's alpha coefficient of 0.931.

### Brief Fear of Negative Evaluation Scale(BFNES)

BFNES was developed by Prof. Leary in 1983 and has 12 items in total, including 4 reverse scoring items and 8 positive scoring items, and a Cronbach's alpha coefficient of 0.90 [42]. A five-point Likert scale was used, with higher scores meaning that individuals were more afraid of receiving negative evaluations. Studies by Rodebaugh and Weeks have shown that reverse-scored items may elicit erroneous and confounded responses from subjects, thereby decreasing subjects' true level of fear of negative evaluations [43, 44]. The BFNES, which retains only positive scoring questions, has been validated in a Chinese population [45], so this study wanted to use the BFNES, which retains only positive scoring questions, to measure college students' negative evaluative fears. In this study, the Cronbach's alpha coefficient for this scale was 0.949.

### Physical Appearance Perfectionism Scale(PAPS)

PAPS was first developed by Chinese scholar Zhang Xiaoyan in 2007 and later adapted by Hongfei Yang in 2012 with a Cronbach's alpha coefficient of 0.88 [34]. The scale has 13 items and is scored on a five-point Likert scale from "Strongly Disagree" to "Strongly Agree". The greater the score, the stronger the individual's physical perfectionism in appearance. For this scale in the study, the Cronbach's alpha coefficient was 0.915.

### Statistical analysis

Statistical analysis was performed using IBM SPSS version 27.0 and AMOS version 24.0 (IBM Corporation). Categorical variables were expressed as percentages (%), while continuous variables were expressed as mean ± standard deviation (SD). The rand, rank, and roundup functions in Excel were used to randomly divide the 2374 participants into two groups, the first group (N_1_ = 1187) was used for the reliability and validity test of the Chinese version of SESMEB, and the second group (N_2_ = 1187) was used to analyze potential influences on the effects of social media on eating behavior. Multiple stepwise linear regression analysis was used to explore the potential influences of social media on eating behavior.

#### Item analysis

The Critical Ratio Decision Value (CR) method and correlation analysis were used to analyze the scale items. The Critical Ratio Decision Value method was used to rank the total scores of the SESMEB scale from high to low, with the top 27% as the high group and the bottom 27% as the low group. The t-value obtained from the independent samples t-test for the high and low subgroups was used as the decision value (CR) to evaluate the differentiation of the items, and the items with a CR value of less than 3 and a statistically insignificant difference were excluded [46]. Using Pearson correlation analysis, the correlation coefficients between the overall scores on the scale and the scores on each item were determined, to assess the degree of agreement of their measurement attributes, and to exclude the items with correlation coefficients less than 0.4 [47].

#### Validity analysis

##### Content validity

Two dietary experts and five medical experts were invited to rate each item of the scale on a 4-point scale (1–4 for " no relevance", " low relevance", " strong relevance", and " very strong relevance"). The content validity of the scale was evaluated by content validity index (CVI) [48], including item-level content validity index (I-CVI), universal agreement scale-level content validity index (S-CVI/UA), and average scale-level content validity index (S-CVI/Ave). When I-CVI ≥ 0.78, S-CVI/UA ≥ 0.8, and S-CVI/Ave ≥ 0.9, the scale has good content validity [49].

##### Construct validity

Exploratory factor analysis (EFA) and confirmatory factor analysis (CFA) were used to test the construct validity of the Chinese version of the SESMEB. The rand, rank, and roundup functions in Excel were used to randomly divide the data of the first group (N_1_ = 1187) into sample 1 and sample 2 for EFA (n_1_ = 594) and CFA (n_2_ = 593), respectively.

In Sample 1 (n_1_ = 594), the Kaiser–Meyer–Olkin (KMO) test [50] and Bartlett's test of sphericity [51] were used to determine suitability for factor analysis. Data were deemed appropriate for factor analysis when the KMO value was higher than 0.60 and Bartlett's test of sphericity was significant (*p* < 0.05). Principal Component Analysis (PCA) and Maximum Variance Orthogonal Rotation were used to assess the internal structure of the Chinese version of SESMEB [52]. Maximum Variance Orthogonal Rotation is the most commonly used orthogonal technique, which minimizes the complexity of the factors while maximizing the variance of factor loading [53]. Factors are extracted based on eigenvalues, cumulative variance contribution, and Scree plot.

In Sample 2 (n_2_ = 593), CFA was used to assess the fit of the structural model [54]. This study assessed the following indexes: Chi-square (χ^2^) and degrees of freedom (df), root mean square error of approximation (RMSEA), root mean square residual (RMR), goodness-of-fit index (GFI), tucker-lewis index (TLI), comparative fit index (CFI), and incremental fit index (IFI) [55]. A model with χ^2^/df < 3, RMSEA, and RMR < 0.08 [56], and a GFI, TLI, CFI, and an IFI > 0.90 is considered to be reasonably well fitted [57]. Measurement invariance of the model was further tested by sociodemographic characteristics gender, ethnicity, education, and major using a multigroup CFA. Next, the standardized factor loadings were used to calculate the average variance extracted (AVE) and CR values. The model's convergent validity was assessed using the AVE values, and its composite reliability was assessed using the CR values. The AVE values were greater than 0.5 for good convergent validity, and the CR values were greater than 0.7 for good composite reliability [58]. Finally, the correlation coefficients and √AVE of each item output according to AMOS 24.0 were used to judge the model's discriminant validity. If √AVE is greater than the correlation coefficients between each item, the discriminant validity of the model is good.

##### Criterion-related validity

In the present study, Pearson correlation analysis was used to conduct a preliminary inference about the validity of the Chinese version of the SESMEB, using the External Eating scale as a standardized instrument.

#### Reliability analysis

The internal consistency of the scale was assessed using McDonald's Omega coefficient, Cronbach's alpha coefficient, and split-half reliability. Cronbach's alpha coefficient was also calculated after deleting each item to see if deleting an item improved the scale's internal consistency. The fold-half reliability was calculated by dividing the scale into two parts based on the item order, and the correlation coefficient of the two parts was calculated by using Spearman correlation analysis. McDonald's Omega coefficient is considered one of the best choices for calculating reliability [59, 60], with values of 0.8 or higher indicating good internal consistency [61]. Cronbach's alpha coefficient and fold-half coefficient ≥ 0.70 were regarded as good internal consistency [62].

Intraclass correlation coefficient (ICC), that is, test–retest reliability, was used to assess the scale's stability. Two weeks after the completion of the first test, 50 college students who had left their contact information were asked to fill out the Chinese version of the SESMEB again. Spearman correlation analysis was used to assess the correlation between the two tests. If the correlation coefficient was greater than 0.7, test–retest reliability was good [63].

## Results

### Demographic characteristics

The survey included 2374 college students, of which 428 (18.0%) were male and 1946 (82.0%) were female. The mean age was 19.48 years (SD:1.70, age range 18–30 years). Undergraduates comprised the majority (53.9%) of our study, junior college students comprised 39.6% of the total, and a small number were postgraduates (6.4%). The study population was dominated by first and second-year students (86.6%), with medical specialties comprising more than four-fifths (87.2%) of the study population.1422 (59.9%) of the respondents indicated that they used social media often or always. 575 (24.2%) of the respondents in this study had a BMI of overweight or obese, and 560 (23.6%) had a thin BMI. Further characteristics of the demographics are detailed in Table 1.

**Table 1:** Demographic characteristics (*N* = 2374)

Variable	Total (N%)
Gender	
Male	428(18.0)
Female	1946(82.0)
Grade (academic year)	
First	1282(54.0)
Second	774(32.6)
Third	234(9.9)
Fourth	46(1.9)
Fifth	38(1.6)
Education	
Junior college students	941(39.6)
Undergraduates	1280(53.9)
Postgraduates	153(6.4)
Major	
Non-medical	305(12.8)
Medical science	2069(87.2)
Ethnicity	
Han	2070(87.2)
Ethnic minority	304(12.8)
Religious beliefs	
With	2257(95.1)
Without	117(4.9)
Only child	
Yes	1406(59.2)
No	968(40.8)
Social media use frequency	
Never	74(3.1)
Seldom	389(16.4)
Sometimes	489(20.6)
Often	899(37.9)
Always	523(22.0)
BMI	
BMI < 18.5	560(23.6)
18.5 ≤ BMI < 24	1239(52.2)
24 ≤ BMI < 28	425(17.9)
BMI ≥ 28	150(6.3)

### Cultural adaptation in SESMEB

Based on the opinions of experts and the feedback from college students who initially finished the scale, the scale was modified in this study with the permission of the original authors. Item 6 "I follow nutrition news/blogs/pages on social media" was modified to "I follow nutrition-related TikTok/Weibo/ RED bloggers on social media". The most common social media used by Chinese college students are TikTok, Weibo, and RED, and very few of them follow news, blogs, and pages. Therefore, the 6th item was modified, aiming to make the content of the scale more consistent with the real circumstances in China, so that the participants could better comprehend and respond to the questions, to ensure the applicability and accuracy of the scale. Table 2 shows the mean (SD) scores of all participants for each item in the revised Chinese version of the SESMEB.

**Table 2:** Mean (SD) scores of participants for each SESMEB item on the Chinese revision (*N* = 2374)

Items on Chinese Revision of the SESMEB	Mean (SD)
(1)Inclusion of a food on social media influences my view of that food	2.68(0.95)
(2)I see and consume any food on social media that are not my food habit	2.25(0.89)
(3)Even though I'm full, I eat a food/dish I see on social media	2.47(1.04)
(4)I think that the foods on social media are more beneficial for health	2.24(0.85)
(5)After I started using social media, my fast-food/cook-chill food consumption increased	2.53(0.95)
(6)I follow nutrition-related TikTok/Weibo/RED bloggers on social media	2.68(0.93)
(7)Without getting tired I buy/cook a food/dish that I see on social media	2.36(0.93)
(8)I regulate my diet according to shared news/photos/videos about the foods/dishes I see on social media	2.43(0.92)
(9)I am constantly snacking when surfing on social media, and I realize how much I've eaten later	2.38(0.95)
(10)I am interested in foods/dishes shared by celebrities on social media and I consume that food/dish	2.34(0.93)
(11)If I did not use social media, my time for eating would be reduced	2.31(0.93)
(12)When surfing on social media, even though I am full I am snacking	2.46(0.98)
(13)I consume foods/dishes shared by people who have a lot of followers on social media	2.34(0.93)
(14)I think foods/dishes with more like/share on social media are healthier	2.28(0.97)
(15)The foods/dishes that I see on social media arouse my desire to eat	2.86(0.95)
(16)I consume foods/dishes with more news/photo/video likes on social media	2.46(0.97)
(17)I think that foods/dishes with more like/share on social media are more reliable	2.29(0.97)
(18)On the days I use social media for a long time, my desire to eat increases, and I eat more	2.84(0.94)

### Item analysis

The thresholds of the Chinese version of the SESMEB total score in the high and low groups were 52 (332, 27.97%) and 39 (326, 27.46%), respectively. The difference between each item in the high and low groups was statistically significant (*p* < 0.001), and its CR value ranged from 28.80 to 39.22 **(**Table 3**)**, which had good differentiation and could effectively measure the response level of different participants. The correlation coefficients between each item and the total score of the scale ranged from 0.730 to 0.831 (*p* < 0.001), which were all higher than 0.4 [47] **(**Table 3**)**.

**Table 3:** Comparison of scores between low-score and high-score groups and correlation between items and overall scores (N_1_ = 1187)

Item	Low-score group (*n* = 332)		High-score group (*n* = 326)		CR	r	*P-***value**
	Mean	SD	Mean	SD			
1	1.74	0.49	3.42	0.74	34.63	0.762	< 0.001
2	1.49	0.53	2.94	0.71	29.55	0.730	< 0.001
3	1.57	0.54	3.35	0.79	33.72	0.777	< 0.001
4	1.51	0.53	2.93	0.72	28.80	0.749	< 0.001
5	1.63	0.52	3.33	0.75	33.67	0.783	< 0.001
6	1.70	0.49	3.34	0.72	34.27	0.767	< 0.001
7	1.51	0.51	3.19	0.76	33.44	0.799	< 0.001
8	1.56	0.52	3.23	0.73	33.84	0.794	< 0.001
9	1.50	0.52	3.21	0.74	34.32	0.783	< 0.001
10	1.51	0.53	3.27	0.71	36.16	0.807	< 0.001
11	1.46	0.51	3.10	0.71	33.94	0.777	< 0.001
12	1.57	0.52	3.31	0.77	33.94	0.784	< 0.001
13	1.52	0.54	3.25	0.71	35.39	0.804	< 0.001
14	1.35	0.50	3.23	0.74	38.30	0.831	< 0.001
15	1.82	0.41	3.55	0.68	39.22	0.800	< 0.001
16	1.50	0.51	3.34	0.74	37.29	0.827	< 0.001
17	1.35	0.48	3.24	0.78	37.79	0.831	< 0.001
18	1.81	0.45	3.52	0.68	38.34	0.807	< 0.001

### Validity analysis

#### Content validity

The content validity of the Chinese version of SESMEB was assessed using the expert evaluation method [64]. The results of the content validity analysis of the Chinese version of the SESMEB showed the I-CVI is 0.86–1, the S-CVI/UA is 0.94, and the S-CVI/Ave is 0.99. The experts were generally in agreement that the scale's items and overall content met the objectives it was designed to measure.

#### Construct validity

##### Exploratory factor analysis (EFA)

**Fig. 1:**
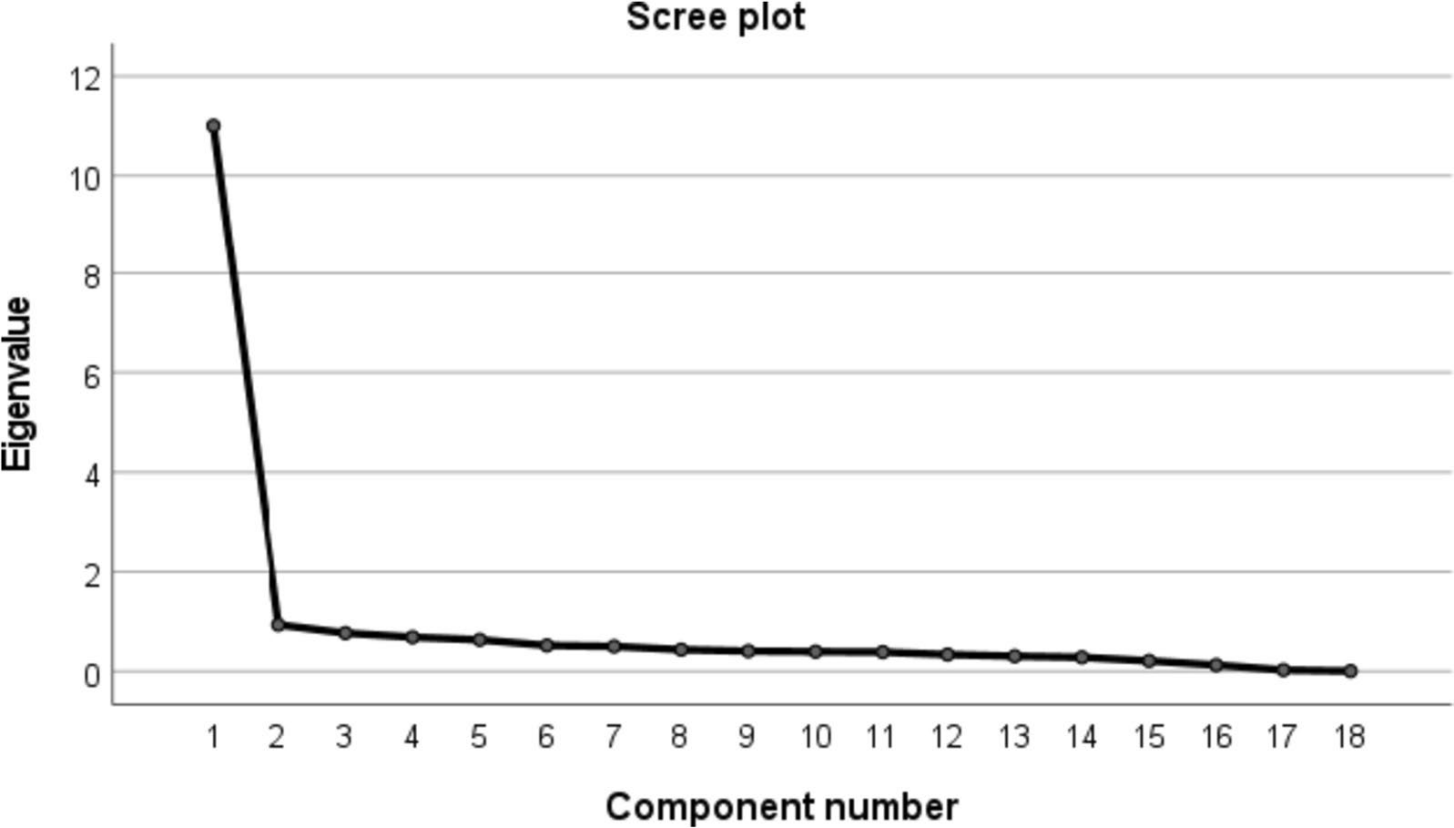
Scree plot of exploratory factor analysis for the Chinese version of SESMEB(n_1_ = 594)

Before EFA analysis, the factoriality of the matrix of sample 1 (n_1_ = 594) was first examined. The Bartlett's test of sphericity was statistically significant (χ^2^ = 11,392.413, *P* < 0.001) and the KMO value was 0.944, which was greater than the minimum acceptable value of 0.6 [50], indicating that there was sufficient correlation between the variables to be suitable for factor analysis. EFA was conducted using PCA and Maximum Variance Orthogonal Rotation, and a total of one common factor with eigenvalue greater than 1 was extracted, with a cumulative variance contribution rate of 61.146%, and the loadings of each item on this dimension were all greater than 0.40. This indicates that all the items in the Chinese version of the SESMEB are pointing to the same latent variable, which is uni-dimensional and has good construct validity. The SESMEB EFA results are shown in Table 4. The structure of the one factor is further explained by the fact that after the second point, the descending tendency of the scree plot becomes less obvious (Fig. 1).

**Table 4:** SESMEB exploratory factor analysis results (n_1_ = 594)

Item	Factor loading	Eigenvalue	Initial Eigenvalue Accumulation(%)	Extract the Sum of Squared Loads(%)
1	0.744	11.006	61.146	61.146
2	0.715	0.938	66.357	
3	0.749	0.771	70.638	
4	0.747	0.688	74.462	
5	0.766	0.636	77.993	
6	0.759	0.522	80.895	
7	0.782	0.503	83.688	
8	0.777	0.438	86.120	
9	0.766	0.408	88.389	
10	0.790	0.398	90.599	
11	0.765	0.389	92.758	
12	0.764	0.339	94.643	
13	0.806	0.307	96.347	
14	0.842	0.283	97.920	
15	0.806	0.212	99.098	
16	0.829	0.128	99.809	
17	0.841	0.028	99.964	
18	0.813	0.006	100.000

##### Confirmatory factor analysis(CFA)

**Fig. 2:**
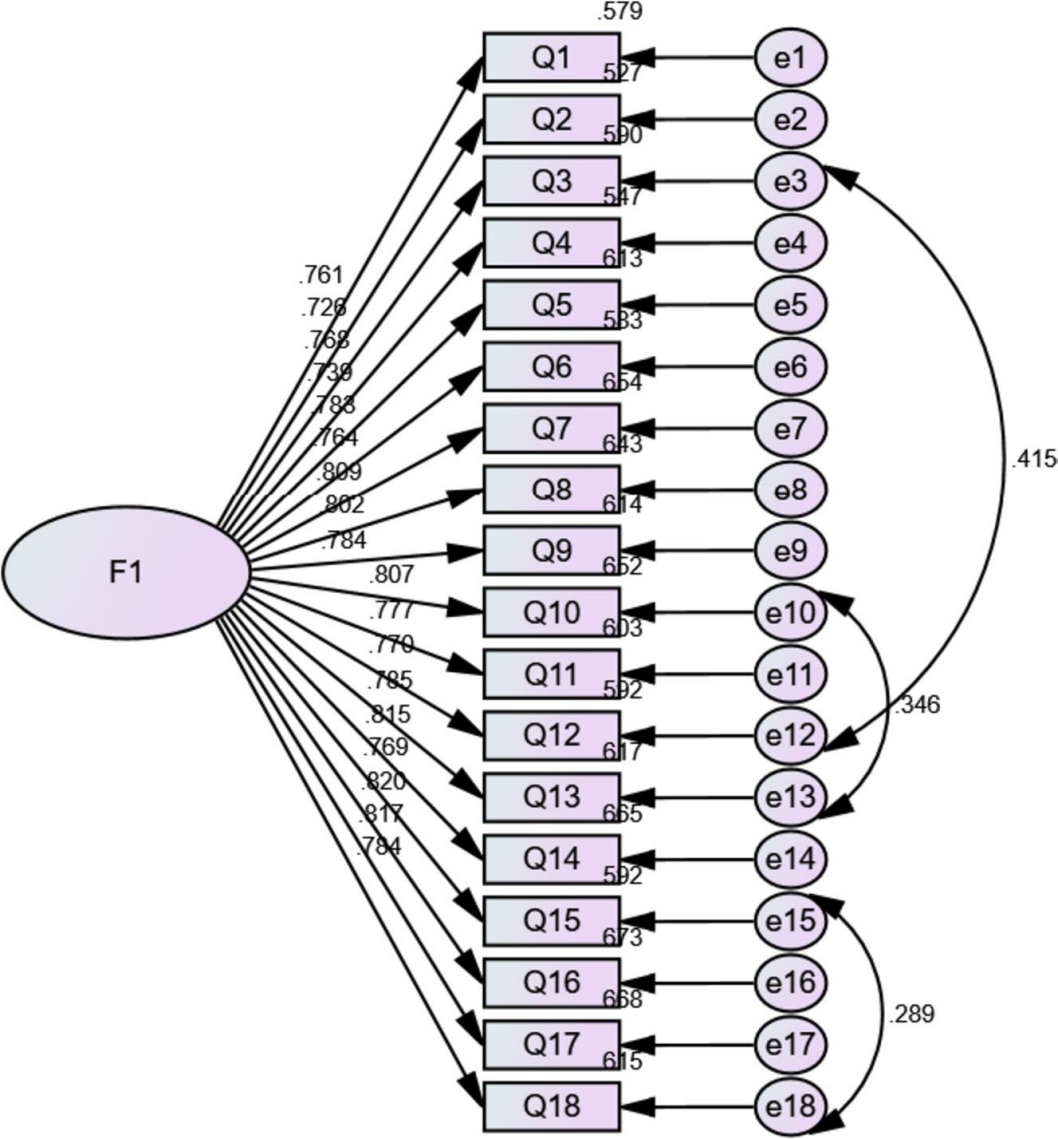
Standard one-factor structural model for the Chinese version of SESMEB(n_2_ = 594)

Further CFA was performed on the Sample 2 (n_2_ = 593) to confirm the EFA-derived model. The fit indicators of the model met the fitness criteria. Results are shown in Table 5. After constructing the correlations of item 3 with item 12, item 10 with item 13, and item 15 with the residual variables associated with item 18 based on the correction index, the data model of the one-factor, 18-item structure fitted satisfactorily, indicating that the model was valid in measuring the latent variables. The graphical expression of the CFA is shown in Fig. 2. The factor loadings for each item varied from 0.726 to 0.820.

**Table 5:** Goodness-of-fit indexes of the one-factor model for the Chinese version of SESMEB (n_2_ = 593)

*χ*^2^	df	*χ*^2^**/df**	IFI	GFI	CFI	TLI	RMR	RMSEA
375.269	132	2.843	0.972	0.931	0.972	0.967	0.020	0.056

##### Measurement invariance test

The results of the tests of measurement invariance of the model by gender, ethnicity, education, and major using multigroup CFA were presented in Table 6. It was shown that the baseline model provided a good fit across gender (χ^2^ /df = 4.93, CFI = 0.882, RMSEA = 0.082), ethnicity (χ^2^ /df = 4.94, CFI = 0.880, RMSEA = 0.082), education (χ^2^ /df = 3.87, CFI = 0.872, RMSEA = 0.070), and major (χ^2^ /df = 3.782, CFI = 0.916, RMSEA = 0.069). These results suggested an equivalent factor structure among the critical demographic variables and provided a baseline model for further tests of measurement invariance. According to previously established criteria, the model had measurement invariance when ΔCFI < 0.010 and ΔRMSEA < 0.015 [65]. The four socio-demographic characteristics of the Chinese version of the SESMEB showed measurement invariance on the measurement weights model, the structural covariances model, and the measurement residuals model, suggesting that the Chinese version of the SESMEB had the same factor structure, factor loadings, and indicator intercepts for gender, ethnicity, education, and major, which can be used to assess and compare the effects of these demographic variables on social media users' eating behavior change.

**Table 6:** Measurement invariance of the Chinese version of the SESMEB across gender, ethnicity, education, and major (n_2_ = 593)

	Model	χ^2^	Δχ^2^	df	Δdf	*P*	CFI	ΔCFI	RMSEA	ΔRMSEA
Gender	Unconstrained	1331.481		270			0.882		0.082	
(n_2_ = 593)	Measurement weights	1342.734	11.253	287	17	0.843	0.883	0.001	0.079	-0.003
	Structural covariances	1342.740	11.259	288	18	0.883	0.883	0.001	0.079	-0.003
	Measurement residuals	1350.444	18.963	306	36	0.991	0.884	0.002	0.076	-0.006
Ethnicity	Unconstrained	1333.252		270			0.880		0.082	
(n_2_ = 593)	Measurement weights	1363.120	29.868	287	17	0.027	0.879	-0.001	0.080	-0.002
	Structural covariances	1367.458	34.206	288	18	0.012	0.878	-0.002	0.080	-0.002
	Measurement residuals	1399.351	66.099	306	36	0.002	0.877	-0.003	0.078	-0.004
Education	Unconstrained	1565.216		405			0.872		0.070	
(n_2_ = 593)	Measurement weights	1603.280	38.064	439	34	0.29	0.872	0.00	0.067	-0.003
	Structural covariances	1606.650	41.434	441	36	0.246	0.872	0.00	0.067	-0.003
	Measurement residuals	1729.166	163.950	477	72	0.00	0.863	-0.009	0.067	-0.003
Major	Unconstrained	1013.482		268			0.916		0.069	
(n_2_ = 593)	Measurement weights	1039.903	26.421	285	17	0.067	0.915	-0.001	0.067	-0.002
	Structural covariances	1044.331	30.849	286	18	0.03	0.914	-0.002	0.067	-0.002
	Measurement residuals	1077.606	64.124	304	36	0.003	0.913	-0.003	0.066	-0.003

##### Convergent validity and discriminant validity

As shown in Table 7, the model had an AVE value of 0.613, which was greater than 0.5, and a CR value of 0.966, which was greater than 0.7, indicating that it had good convergent validity and composite reliability. The √AVE value of 0.783 was greater than the correlation coefficients of each item of the scale (0.536 to 0.761, *p* < 0.001), indicating that the model had good discriminant validity.

**Table 7:** Convergent validity and discriminant validity of the Chinese version of the SESMEB (n_2_ = 593)

Item			Standardized Factor Load	CR	AVE	√AVE
1	< –-	F1	0.761	0.966	0.613	0.783
2	< –-	F1	0.726			
3	< –-	F1	0.768			
4	< –-	F1	0.739			
5	< –-	F1	0.783			
6	< –-	F1	0.764			
7	< –-	F1	0.809			
8	< –-	F1	0.802			
9	< –-	F1	0.784			
10	< –-	F1	0.807			
11	< –-	F1	0.777			
12	< –-	F1	0.770			
13	< –-	F1	0.785			
14	< –-	F1	0.815			
15	< –-	F1	0.769			
16	< –-	F1	0.820			
17	< –-	F1	0.817			
18	< –-	F1	0.784			

#### Criterion-related validity

Correlation analysis was used to describe the correlation between the total score of SESMEB and the total score of External Eating in the sample population of the first group (N_1_ = 1187). The correlation coefficient was 0.609, indicating that the External Eating subscale can provide preliminary inferences about the validity of the Chinese version of the SESMEB.

### Reliability analysis

#### Internal consistency reliability

The results of the reliability analysis showed that the Chinese version of the SESMEB (18 items) had good internal consistency. The McDonald's Omega coefficient and Cronbach's alpha coefficient were 0.964, which was higher than Cronbach's alpha coefficient of 0.928 for the original scale [24]. In addition, Table 8 shows that deleting any of the items individually results in a decrease in the overall Cronbach's alpha coefficient. The split-half reliability coefficient of the scale was 0.953, which had a high level of internal consistency.

**Table 8:** Cronbach's alpha coefficients after deleting specific items in the Chinese version of SESMEB (N_1_ = 1187)

Item	Cronbach's alpha coefficient after deleting a specific item
1	0.963
2	0.963
3	0.963
4	0.963
5	0.963
6	0.963
7	0.962
8	0.962
9	0.963
10	0.962
11	0.963
12	0.963
13	0.962
14	0.962
15	0.962
16	0.962
17	0.962
18	0.962

#### Test–retest reliability

Fifty participants were invited to fill out the questionnaire once more after two weeks. The results showed that the Spearman correlation coefficient between the two tests was 0.849, which is greater than 0.7. This indicates that the Chinese version of the SESMEB has good stability over time.

### Analysis of potential influences on the effects of social media on eating behavior (N_2_ = 1187).

The mean total SESMEB score of 1187 Chinese college students was 44.16 ± 11.23. Multiple stepwise linear regression analyses showed that female, highly educated, non-medical, frequent social media use, online sexual objectification experiences, fear of negative evaluations, and physical appearance perfectionism were potential influences on SESMEB. These seven variables explained 73.8% of the variance in the effects of social media on Chinese college students' eating behavior (Table 9).

**Table 9:** Multiple stepwise linear regression model for overall SESMEB score (N_2_ = 1187)

	B	β	t	*P*	95% CI	Collinearity statistics		Model R^2^
						Tolerance	VIF	
Constant	9.503		8.831	< 0.001	[7.392,11.614]			0.738
Social media use frequency	6.812	0.664	37.498	< 0.001	[6.455,7.168]	0.703	1.421	
Online Sexual Objectification Experiences	0.497	0.223	13.117	< 0.001	[0.423,0.572]	0.765	1.307	
Fear of negative evaluations	0.092	0.064	3.198	< 0.001	[0.035,0.148]	0.559	1.789	
Education	0.969	0.052	3.386	< 0.001	[0.407,1.530]	0.951	1.052	
Physical appearance perfectionism	0.059	0.050	2.567	0.010	[0.014,0.103]	0.580	1.723	
Gender	-1.421	-0.049	-3.191	< 0.001	[-2.294,-0.547]	0.942	1.061	
Major	-1.661	-0.047	-3.052	0.002	[-2.728,-0.593]	0.914	1.094	

## Discussion

In this study, the first attempt was made to introduce the SESMEB scale into China to measure the effects of social media on eating behavior. Subsequently, the Chinese version of the SESMEB was developed through a rigorous cultural adaptation to the translation and psychometric characterization of the scale. Finally, the Chinese version of the SESMEB retained all 18 items of the original version and had good internal consistency reliability. Regarding validity, factor analysis supported having a highly loaded item one-factor structure with good construct validity. This study confirms the validity of the SESMEB scale in measuring the effects of social media on eating behavior and also provides a validated and effective instrument for assessing eating behavior change in the social media environment among Chinese college students. It also confirms the hypothesis that the effects of social media on eating behavior are related to socio-demographic characteristics and that subjects with online objectification experiences, fear of negative evaluations, and appearance perfectionism use social media to have a greater impact on eating behavior.

### The Chinese version of SESMEB has good validity and reliability

Identical to the original scale structure, a one-factor structure was extracted through EFA, explaining 61.146% of the total data variance. The factor loadings of the 18 items on this dimension ranged from 0.715 to 0.842. This indicates that all the items in the Chinese version of the SESMEB point to the same underlying trait, and the scale is unidimensional in character, which can more centrally respond to the actual situation of the effects of social media use on the eating behavior of Chinese college students. CFA showed that the model fit indices all met acceptable standards. The Chinese version of the SESMEB items fit well with this one-factor structural model. The convergent validity, composite reliability, discriminant validity, content validity, and criterion-related validity of the model were all good. The Chinese version of SESMEB can make a preliminary judgment on the effects of social media on eating behavior. The internal consistency coefficients of the Chinese version of SESMEB satisfy the requirements. The value of McDonald's Omega coefficient reflects the internal consistency of the instrument more consistently than the value of Cronbach's alpha coefficient because it corrects for the underestimation bias of Cronbach's alpha when the tau-equivalence assumption is violated [66]. The McDonald's Omega coefficient in this study was 0.964, indicating a high correlation between each item and the total score of the Chinese version of the SESMEB. The test–retest reliability of the scale is good, and the Chinese version of the SESMEB can stably measure the influence of social media on Chinese college students' eating behavior.

In addition, the Chinese version of the SESMEB did not differ between gender, ethnicity, education, and major according to measurement invariance tests of measurement weights model, structural covariances model, and measurement residuals model. This suggests that the scale structure is generally applicable to individuals within these socio-demographic variables, that is, the scale provides a stable and undifferentiated measure of eating behavior change that occurs when Chinese college students use social media.

### For constructing the explanation of the correlation between the residual variables among the items

The results of many studies have shown that residual correlation between items is allowed to improve model fit [67–69], especially when the correlation between measurement errors is unavoidable [70]. First, this study conducted a residual correlation between item 3 and item 12. The correlation coefficient was 0.415, and both items describe the effects of social media on the eating behavior of satiated individuals, so we consider the residual correlation between item 3 and item 12 to be reasonable. Second, we conducted item 10 and item 13 with a residual correlation and a correlation coefficient of 0.346. Both items describe how food shared by social media celebrities can have an impact on individuals' eating choices, so we consider the residual correlation between item 10 and item 13 to be reasonable. Finally, we conducted a residual correlation between item 15 and item 18 with a correlation coefficient of 0.289. Both items describe the effects of social media on individuals' desire to eat. The residual correlation between item 15 and item 18 is considered reasonable. The CFA model with residual correlation has been widely applied [71, 72]. Therefore, the model has been carefully formulated to maximize its correspondence with the real model.

### Analysis of potential influencing factors of social media's effects on Chinese college students' eating behavior

Using the validated Chinese version of the SESMEB, we confirmed that the frequency of social media use, OSOES scores, BFNES scores, and education were the top four influences on the SESMEB (based on the standardized coefficient β). Among them, individuals with more frequent social media use, more online objectification experiences, more severe fear of negative evaluations, and higher education had more intense expressions of SESMEB. Our findings are also confirmed by the existing literature. One study showed that 97.5% of young people regularly use at least one social media platform [73]. Nowadays, online media is filled with images, videos, and texts about the ideal body and perfect appearance, and young people who spend a significant amount of time on the Internet every day are unavoidably influenced by these messages, leading to dissatisfaction with their own body appearance and changes in eating behavior. Excessive use of cell phones can also lead to a range of psychological problems, including shyness, low self-esteem, anxiety, and depression [74], and psychological problems are in turn closely related to eating disorders [75, 76], which may well explain the more severe expression of SESMEB in individuals with high social media use. Relevant literature suggests that online objectification experiences can have a direct impact on restrictive eating behaviors [27]. Online objectification experiences, as a source of life experience and external environmental stimuli, were significantly and positively correlated with college students' body shame. When individuals suffer from objectifying experiences online, they are more inclined to compare their bodies with the social standard of ideal beauty, and become dissatisfied with their bodies, resulting in changes in eating behaviors [77]. Fear of negative appearance evaluations affects an individual's eating attitudes [78, 79]. On the one hand, Levinson's study suggests that fear of negative evaluations of appearance is a risk factor for eating disorders and that treating the fear of negative appearance evaluations may alleviate eating disorders [80]. On the other hand, there is also evidence in the literature that individuals with eating disorders may be more concerned that their appearance will be evaluated negatively. Compared to a control group, a clinical sample of women suffering from eating disorders expressed a greater fear of negative evaluation [81]. Both the experience of online objectification and the fear of negative evaluation are inextricably linked to the image of the ideal slimmed-down body disseminated on social media. In our study, the eating behavior of highly educated individuals was more susceptible to being influenced by social media, unlike previous studies [82]. In today's society, against the background of expanding enrollment and gradually improving the quality of training, postgraduates are facing academic pressure, employment pressure, economic pressure, and so on. This prompts them to look for opportunities to vent their frustrations on social media, excessive consumption of food and shopping is one of the ways for contemporary college students to alleviate their stress. In addition, it has been reported that postgraduates are more likely to suffer from mental health problems, with more than one-third of postgraduates suffering from depressive symptoms [83], and that depressed mood is a risk factor for body dissatisfaction [84], and that body dissatisfaction in an individual will lead to unhealthy eating behaviors. The above evidence supports the results obtained in this study.

Our study also showed that PAPS scores, gender, and major were other important risk factors for SESMEB. According to our research, female college students' eating behavior was more significantly affected by social media, and Wilksch's study also showed that women's eating disordered behaviors as a result of social media were nearly one-third higher than men's [85]. Nelson had the same finding [86], as female peers were more likely to talk about appearance than male peers. The ideal body disseminated in the mass media increases the likelihood that women will become unhappy with their bodies. Peers and media images are the most common and significant targets for body and appearance comparisons among adolescent girls and young adult women and men [87, 88]. Females are more likely to be exposed to a media environment that emphasizes "thinness" as beauty and are more likely to compare their body shape and appearance to the social media ideal of a slimmer body with a perfect appearance [89]. This study found that medical students' eating behavior was less prone to the influence of social media compared to other majors. The reason for this difference may be that medical students have higher health literacy, pay more attention to eating healthy, and are less likely to be disturbed by information published in the media. Some studies have shown that professional education in medical specialty courses has a significant effect on health literacy [90], which may explain why medical students' eating behavior is less susceptible to the influence of social media. A plausible explanation for the positive correlation between physical appearance perfectionism and SESMEB is that excessive use of social networking sites may increase college students' tendency towards appearance perfectionism, while the tendency towards appearance perfectionism can also increase the risk of eating disorders among college students [91, 92]. Martini's study also demonstrated that perfectionism and excessive attention to the body can lead to disordered eating behaviors in individuals [37].

### Limitations

Firstly, this is a cross-sectional study and no causal inferences can be made. Secondly, all data were self-reported. However, participants were unlikely to fill in incorrect information as they were prompted to respond anonymously. Thirdly, the ease of sampling limits the generalizability of the conclusions, so future validation should be conducted on a broader population. Lastly, it is hard to compare the results with other studies because of the inadequate literature on SESMEB research.

## Conclusions

The Chinese version of the SESMEB scale contains 18 items and supports a one-factor structure with satisfactory reliability and validity. Therefore, the validated Social Media Effects on Eating Behaviour Scale is more suitable for Chinese people. Future studies should further explore its suitability to other populations and analyze the underlying influences on eating behavior change in the social media environment. In addition, initial screening and identification of individuals who are female, highly educated, non-medical, have frequent social media use, online sexual objectification experiences, fear of negative evaluations, and physical appearance perfectionism can help to identify eligible high-risk populations during the development of public health intervention strategies, to popularize them early to reduce the health hazards and economic losses from irrational use of social media.

## Data Availability

No datasets were generated or analysed during the current study.

## Abbreviations

SESMEB: The Scale of Effects of Social Media on Eating Behaviour
DEBQ: Dutch Eating Behaviour Questionnaire
OSOES: Online Sexual Objectification Experience Scale
BFNES: Brief Fear of Negative Evaluation Scale
PAPS: Physical Appearance Perfectionism Scale
EFA: Exploratory Factor Analysis
CFA: Confirmatory Factor Analysis
PCA: Principal Component Analysis
RMSEA: Root Mean Square Error of Approximation
RMR: Root Mean Square Residual
GFI: Goodness-of-Fit Index
CFI: Comparative Fit Index
IFI: Incremental Fit Index
TLI: Tucker-Lewis Index
AVE: Average Variance Extracted
